# Description of Plasma Penicillin G Concentrations after Intramuscular Injection in Double-Muscled Cows to Optimize the Timing of Antibiotherapy for Caesarean Section

**DOI:** 10.3390/vetsci8050067

**Published:** 2021-04-21

**Authors:** Salem Djebala, Siska Croubels, Marc Cherlet, Ludovic Martinelle, Damien Thiry, Nassim Moula, Arnaud Sartelet, Philippe Bossaert

**Affiliations:** 1 Clinical Department of Ruminants, Faculty of Veterinary Medicine, University of Liège, Quartier Vallée 2, Avenue de Cureghem 7A-7D, 4000 Liège, Belgium; asartelet@uliege.be (A.S.); p.bossaert@uliege.be (P.B.); 2 Department of Pharmacology, Toxicology and Biochemistry, Faculty of Veterinary Medicine, Ghent University, Salisburylaan 133, 9820 Merelbeke, Belgium; Siska.Croubels@UGent.be (S.C.); Marc.Cherlet@UGent.be (M.C.); 3 CARE-FEPEX Experimental Station, Faculty of Veterinary Medicine, University of Liège, Quartier Vallée 3, Chemin de la Ferme 6, 4000 Liège, Belgium; lmartinelle@uliege.be; 4 Bacteriology, Department of Infectious and Parasitic Diseases, Faculty of Veterinary Medicine, University of Liège, Quartier Vallée 2, Avenue Cureghem 6, 4000 Liège, Belgium; damien.thiry@uliege.be; 5 Department of Veterinary Management of Animal Resources, Faculty of Veterinary Medicine, Fundamental and Applied Research for Animal & Health (FARAH), University of Liège, 4000 Liège, Belgium; nassim.moula@uliege.be; 6 GIGA—Animal Facilities—ULiège—B 34, 4000 Liège, Belgium

**Keywords:** preoperative antibiotic, procaine benzylpenicillin suspension, elective caesarean section, plasma concentration, intramuscular administration

## Abstract

In order to improve the efficacy of penicillin injection during caesarean section, we aimed to identify the optimal timing of its preoperative administration. A study was conducted in 12 adult, non-pregnant Belgian Blue cows. To evaluate the plasma penicillin concentrations, blood samples were taken from the jugular vein at −5, 15, 30, 45, 60, 120, 240, 480 min relative to the intramuscular (IM) injection of 21,000 IU/kg of body weight of penicillin G. Results showed that plasma concentrations at 15 min after IM injection (668.3 ± 73.7 ng/mL) largely exceeded the minimal inhibitory concentration (MIC) of penicillin-sensitive bacteria (MIC < 125 ng/mL). With increasing time, plasma concentrations continued to rise, attaining an increasing proportion of moderately sensitive bacteria (250 ng/mL > MIC < 2000 ng/mL). The maximal concentration was reached between 1 and 4 h (average: 1.495.1 ± 181.7 ng/mL) after IM injection in the majority of cows, and decreased non-significantly to 1002.1 ± 93.2 ng/mL at 8 h. In conclusion, plasma penicillin concentrations at 15 min after an IM injection inhibit penicillin-sensitive bacteria. However, in order to obtain the maximal protective effect of the antibiotherapy, surgery should be started at 1 to 2 h after IM penicillin injection.

## 1. Introduction

Laparotomy is a common surgical procedure in bovine veterinary medicine that is performed for digestive [1,2] and reproductive indications [3,4,5]. In Belgium, (elective) caesarean section (CS) in the double-muscled Belgian Blue cow is a very frequently carried out laparotomy [6,7]. Contamination of the surgical site during CS is practically inevitable [8,9,10] and may be caused by bacteria originating from the environment, the surgeon and his/her materials [10], the incised uterus [8], or haematogenous spread [11]. Since bacterial infection may lead to severe postoperative complications [12,13,14,15], veterinarians systematically administer antibiotics when performing a CS [6]. However, several aspects of antibiotic therapy, including choice of drug, timing and route of administration, and treatment duration, remain the subject of controversy [6].

The goal of prophylactic antibiotic treatment is to reach concentrations that sufficiently reduce bacterial proliferation at the surgical site for the entire duration of the procedure [16,17,18,19]. In CS in women, a prophylactic antibiotic injection before the onset of surgery has been shown to effectively lower the risk of infectious complications, and consequently, has been accepted as a standard protocol [17,18,19].

Although it seems reasonable to adopt this principle in bovine veterinary practice, preoperative antibiotic treatment before CS is not recommended in the current guidelines established by the Belgian Centre of Expertise on Antimicrobial Consumption and Resistance in Animals (AMCRA) [20]. More precisely, in case of a non-complicated elective CS, AMCRA recommends a single dose of antibiotics administered locally during surgery. Preoperative antibiotic treatment is recommended only when serious complications are expected [20]. In a recent survey, Djebala and co-workers [6] reported that only a minority (11%) of Belgian rural veterinarians administer preoperative intramuscular (IM) antibiotics when performing a CS. Veterinarians predominantly administer antibiotics during CS via intraperitoneal injection or injection between the incised muscle layers, and/or after CS with IM injection. Procaine benzylpenicillin (procaine penicillin G) is the most commonly used drug. The fact that treatment habits vary widely and seem to be based on veterinarians’ personal experience rather than on scientific evidence or national recommendations raises questions about these practices, but also about the feasibility of the guidelines themselves.

Assuming that preoperative antibiotics therapy could result in more efficient protection at CS, the ideal timing relative to the onset of surgery remains unclear. National guidelines state that a preoperative IM injection, when indicated, should be administered at least 60 min before the onset of surgery [20], but the scientific justification for this recommendation is unclear. Furthermore, a practical problem arises since veterinarians do not wait 60 min but initiate CS immediately after arrival at the farm. Plasma concentrations of penicillin G after IM injection have previously been reported in cattle [21,22,23,24], but only two studies have reported the plasmatic concentration within the first hour after injection. Additionally, these studies were performed in a limited number of Holstein cows injected in the gluteal muscles [23,24]. It may be erroneous to extrapolate these data to Belgian Blue cows as they have a different body composition than Holstein cows, and usually receive IM treatments in the neck muscles and not in the gluteal muscles because of their high butchery value [7].

Therefore, as a contribution to our attempt to adjust clinical practice to evidence-based guidelines, we hypothesized that antibiotic treatment around CS, the most common laparotomy used in Belgian rural veterinary practice, can be optimized by applying a preoperative, rather than a peri- or postoperative treatment. The aim of this study was to describe the plasma concentrations of penicillin G after injection of procaine benzylpenicillin in the neck muscles of Belgian Blue cows in order to establish the preferred timing of preoperative antibiotic administration.

## 2. Materials and Methods

All procedures received the approval of the Ethical Committee of Liège University (File number 2141).

Twelve non-pregnant, adult, healthy Belgian Blue cows aged between 3.5 and 7 years were selected at the experimental farm of Liège University. The cows had not received any treatment for two months in order to avoid interference with previous procaine penicillin G treatments [25,26]. All cows were weighed (body weights ranged from 550 to 824 kg) and moved to the experimental unit the day before the trial. Cows were housed in a tie-stall with enough space for eating, drinking (water and hay ad libitum), standing and lying down for the entire duration of the trial. The skin at the site of the left jugular vein was shaved and disinfected using povidone-iodine soap (Vet-Clean, 7.5%, Laboratoria Smeets NV, Antwerp, Belgium) and alcohol (Alcohol denature^®^, 96°, VWR Chemicals, Leuven, Belgium). Each cow received a permanent 14G/80 mm catheter (Infraflon2, Ecouen, Paris, France).

A dose of 21,000 IU procaine penicillin G per kg of body weight (Peni-Kel^®^, 300,000 IU/mL, Kela Laboratoria, Hoogstraten, Belgium) was injected in the right neck muscles, in the centre of a triangular area defined by the spine, the neck ligament and the shoulder line [27] with a 20 mL syringe (Henry Schein, Langen, Germany) and a 16G/40 mm needle (Henry Schein, Gillingham, UK). The maximum volume per injection site was 20 mL.

Blood samples were obtained in 10 mL heparinized blood tubes (Vacutainer^®^) (BD, Plymouth, UK) from the catheter at −5, 15, 30, 45, 60, 120, 240 and 480 min relative to procaine penicillin G administration. Before and after each collection, the catheter was flushed with 5 mL of heparinised water (25 IU/mL) (LEO Pharma, Ballerup, Denmark). The first 10 mL of blood were discarded in order to obtain an undiluted sample. Samples were stored at room temperature for 30 min until centrifugation (Hettiche, Sérézin du Rhône, France) at 1029 g for 15 min, and plasma was immediately stored in an Eppendorf cup at −80 °C until analysis.

One week after the trial, samples were dispatched for analysis to the Laboratory of Pharmacology and Toxicology of the Faculty of Veterinary Medicine, Ghent University, Belgium. The penicillin G concentrations were measured using an in-house developed and validated LC-MS/MS method. Sample preparation consisted of a deproteinization step using acetonitrile, followed by a back-extraction of the acetonitrile with dichloromethane. The upper aqueous layer was further diluted 1/10 with Milli-Q water, and a 5 µL aliquot was injected into the LC-MS/MS system. Chromatographic separation of penicillin G and penicillin V (phenoxymethylpenicillin, used as internal standard) was achieved on an Acquity UPLC^®^ BEH C18 1.7 µm 2.1 × 50 mm column (Waters, Zellik, Belgium), in combination with a guard column of the same type, using a gradient elution with 0.1% (*v*/*v*) acetic acid in water and acetonitrile. Components were detected on a Quattro Premier XE triple quadrupole mass spectrometer (Micromass, Waters, Wilmslow, UK) equipped with an ESI (electrospray ionization) ion source operating in the positive ionization mode. Components were detected in MS/MS mode using the following MRM (Multiple Reaction Monitoring) transitions: for penicillin G, *m*/*z* (mass-to-charge ratio) 335.1 > 176.0 (quantifier ion), and *m*/*z* 335.1 > 160.0 (qualifier ion); for penicillin V, *m*/*z* 351.1 > 160.0, all at a collision energy (CE) of 15V. Method validation was performed according to EC (2002/657/EC) and VICH (VICH GL49) guidelines. A validation scheme was performed over 3 days with evaluation of linearity in the range of 25–10,000 ng/mL. Within-day and between-day evaluation of accuracy and precision were evaluated at low, medium, and high concentration levels, that is, 25, 1000, and 10,000 ng/mL, respectively. The limit of quantification (LOQ) of the method was established at the lowest level evaluated for accuracy and precision, i.e., 25 ng/mL. The limit of detection (LOD) of the method on the other hand was as low as 1.7 ng/mL using the S/N = 3 criterion. The validation parameters that were evaluated fulfilled the criteria given in both guidance documents (2002/657/EC, VICH GL49) [28,29].

Plasma samples from cows who had never received procaine penicillin G were used as control samples.

For each cow, the peak plasma concentration (C*max*), the time C*max* was reached (T*max*), the moment and duration at which plasma penicillin G concentrations exceeded 125 and 250 ng/mL, i.e., the minimal inhibitory concentrations (MIC) thresholds for susceptible and intermediately susceptible bacteria, respectively [30], were established.

In order to estimate the most feasible time interval between injection by the veterinarian and the onset of surgery, we followed four experienced rural practitioners during 10 routine CS each. We recorded the time spent on local anaesthesia and preparation of the surgical site, the materials and scrubbing, until the first incision. Furthermore, the time between first incision and skin closure was measured. The timed periods were rounded up or down into full minutes.

Statistical analysis were performed using SAS (2001) (Statistics, Version 8.2. SAS Institute, Cary, NC, USA). Continuous data were checked for normal distribution with the Shapiro–Wilk test. The mean and standard error (Mean ± SE) were established for the plasma penicillin G concentrations at each sampling time, C*max*, time of CS preparation and time of CS realisation. Analysis of variance (Proc, ANOVA) was used to compare the antibiotic concentrations at the different sampling moments. The threshold of significance was defined as *p* < 0.05.

## 3. Results

### 3.1. Plasma Concentrations of Penicillin G

**Figure 1:**
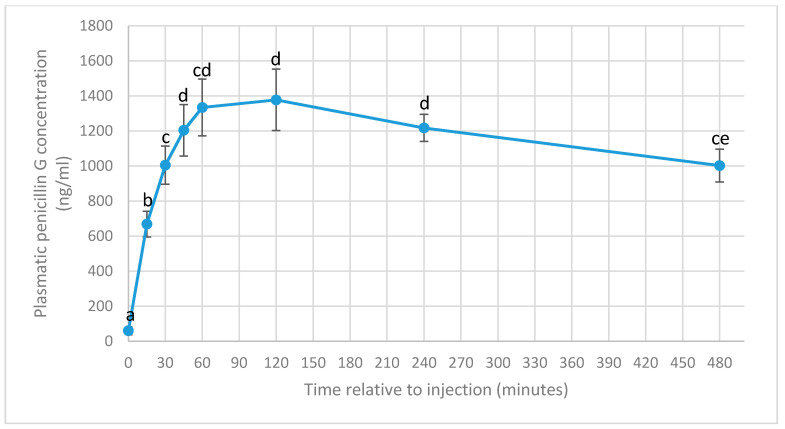
Mean plasma penicillin G concentration (ng/mL) at different sampling times (−5, 15, 30, 45, 60, 120, 240, and 480 min) relative to intramuscular administration of 21,000 IU/kg of procaine benzylpenicillin in 12 Belgian Blue cows. Error bars represent the standard error. a–e values bearing different letters indicate a statistical difference (*p* < 0.05).

Detailed results for the penicillin G plasma concentrations are displayed in Figure 1 and Table 1.

Surprisingly, all cows showed detectable traces of penicillin G at the first sampling (−5 min), ranging from 3.7 ng/mL to 291.9 ng/mL (59.9 ± 22.6 ng/mL).

At 15 min after injection, the mean penicillin G concentration increased significantly (668.3 ± 73.7 ng/mL) compared to the basal level (*p* = 0.0004), ranging from 373.9 to 898.0 ng/mL. At 30 min, plasma penicillin G concentrations further increased significantly (1004.2 ± 108.5 ng/mL) compared to 15 min earlier (*p* = 0.046), ranging from 633.4 ng/mL to 1790.5 ng/mL. Plasma penicillin G concentrations at 45 min (1203.7 ± 146.0 ng/mL) increased non-significantly compared to 15 min earlier (*p* = 0.23), and ranged between 672.0 ng/mL and 2438.5 ng/mL. At 60 min after injection, the mean plasma concentration reached 1333.7 ± 161.6 ng/mL and ranged between 744.4 ng/mL and 2587.2 ng/mL, and were not statistically different from concentrations at 45 min (*p* = 0.43). Two hours after the onset of the trial, penicillin G concentrations varied between 840.4 ng/mL and 2882.5 ng/mL with a mean of 1376.7 ± 175.2 ng/mL, which represented the maximal mean concentration, and were not statistically higher than the values measured at 1 h (*p* = 0.79). At 4 h, the plasma concentrations decreased non-significantly (*p* = 0.33) to 1217.1 ± 76.7 ng/mL, ranging from 843.4 ng/mL to 1660.1 ng/mL. Finally, penicillin G concentrations continued to decline non-significantly (*p* = 0.19) at 8 h after injection to 1002.1 ± 93.2 ng/mL, ranging from 667.8 ng/mL to 1642.6 ng/mL.

The C*max* varied between 918.9 ng/mL and 2882.5 ng/mL with a mean of 1495.1 ± 181.7 ng/mL, and was reached after 1 h in 4 cows, after 2 h in 3 cows, after 4 h in 4 cows and after 8 h in 1 cow (*p* = 0.63).

For the entire duration of the experiment, ranging from 15 min to 8 h after injection, plasma penicillin G concentrations exceeded the thresholds of 125 and 250 ng/mL in all cows.

### 3.2. Duration of Surgery Preparation and Realisation

The time required for CS preparation varied between 7 and 12 min, with a mean and SE of 9.2 ± 0.2 min. The total duration of surgery (from the skin incision until the skin closure) varied between 27 and 36 min with a mean and SE of 31.0 ± 0.4 min.

## 4. Discussion

Since all use of antibiotics stimulates the development of bacterial resistance, veterinarians are encouraged to avoid the use of antibiotics whenever possible and to optimize their efficacy when necessary [31,32]. Since CS is a clean-contaminated surgery [8,10,20] and because bacterial infections can cause severe complications [13,14,15], it seems impractical to avoid antibiotic use. However, there are opportunities to improve the effectiveness of antibiotherapy around CS. Rural veterinarians usually inject antibiotics during CS (after suturing the uterus) or after CS [6] and as a result, cows spend a considerable time in surgery without any antibiotic protection. Preoperative IM administration of antibiotics seems a logical step to improve the effectiveness of antibiotic prophylaxis around CS. Concrete data to define the appropriate timing of injection relative to the operation are lacking.

We studied the plasma concentrations of penicillin G since this is a recommended first-choice product [20] and the most frequently used antibiotic drug in the field [6]. Penicillin G is a time-dependent and bactericide antibiotic with a spectrum covering mainly gram-positive and anaerobic bacteria [33,34]. Although most reports studying the plasmatic concentration of penicillin G after IM administration attribute great importance to C*max* [21,23], a more important parameter for time-dependent antibiotics, such as penicillin G, is the duration of plasma concentration above the MIC of the targeted bacteria [35,36]. This is also the rationale of the current study: in order to optimize antibiotic therapy, plasma penicillin G concentrations should exceed the MIC of a maximal range of targeted bacteria for the entire duration of the surgery [34,35,36,37]. Emphasis was placed on plasma penicillin G concentrations immediately after injection.

Penicillin G susceptible bacteria have an MIC < 125 ng/mL, while moderately susceptible bacteria have an MIC ranging from 250 to 2000 ng/mL [30,35]. In the current study, plasma penicillin G concentrations ranged from 373.9 to 1226.9 ng/mL at 15 min after injection, and we can assume that these concentrations will limit the proliferation of penicillin G susceptible bacteria and a proportion of moderately susceptible bacteria at the surgical site [30,33]. Our data show that veterinarians spend even less than 15 min for preparing their CS (9.2 ± 0.2 min), and one shortcoming of our study is the fact that we did not measure penicillin G levels at 5 and 10 min after injection. Interestingly, in one other study [23], plasma penicillin concentrations also exceeded 125 ng/mL at 5 and 10 min after IM injection. Beyond 15 min after injection, penicillin G concentrations continue to rise and it can be expected that an increasing proportion of bacteria will be affected. Most cows reached C*max* at 1 to 4 h after injection, corresponding well to T*max* reported elsewhere [38]. Our results indicate that experienced veterinarians spend on average 31 min from skin incision to closure. Since penicillin G concentrations remained on a plateau until at least 8 h after injection, preoperative IM administration of procaine penicillin G offers suitable protection during the entire CS. Another shortcoming of the current study is the fact that blood sampling was not continued until 24h after injection, although this information can be found in other studies [22,23,24] and the drug prescription leaflet [38].

**Figure 2:**
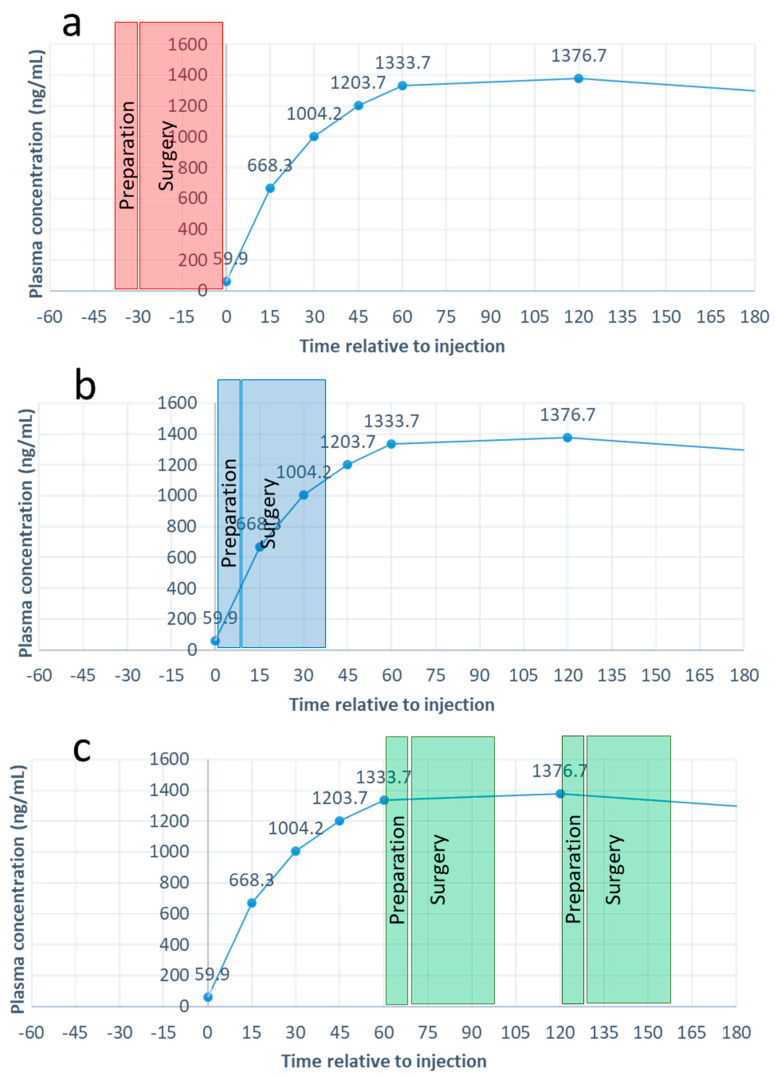
Concentrations of penicillin G during CS in cows based on three different scenarios of timing of penicillin injection relative to preparation and surgery. (**a**) Penicillin is injected during or after CS, as currently recommended and carried out in practice. (**b**) Penicillin is injected by the veterinarian at arrival at the farm. Penicillin G concentrations are rising during surgery but have not yet reached maximal levels. (**c**) Penicillin is injected by the farmer, 1 to 2 h before the vet’s arrival at the farm. Penicillin G concentrations have reached their maximal levels during surgery.

Figure 2 simulates the plasma penicillin concentrations during surgery after IM injection following different scenarios. We can state that penicillin injection by the veterinarian immediately after arrival at the farm will result in plasma concentrations affecting penicillin-sensitive bacteria and an increasing proportion of intermediately sensitive bacteria throughout surgery. This approach is superior to the scenario of perioperative or postoperative injection, as currently recommended (AMCRA) and done in practice [6]. However, in order to obtain maximal effectiveness of the use of penicillin, i.e., maximal penicillin concentrations coinciding with the surgery, the injection should take place 1 to 2 h before the initiation of CS. This is consistent with protocols in human medicine, where antibiotic administration is recommended between 15 min and 2 h before the onset of surgery [16,17,19], and with AMCRA recommendations [20] to perform an IM penicillin injection at least 60 min prior to CS in cows. Since it is unlikely that veterinarians will wait this long before starting surgery after arrival at a farm, maximal penicillin G levels during CS can be obtained if the farmer injects the cow 1 to 2 h before the arrival of the veterinarian, if the legal context allows it.

Plasma penicillin concentrations after IM injection (21,000 IU/kg) of double-muscled cows in the neck are comparable with those found by Conlon et al. [23], who measured penicillin concentrations after a single injection of 20,000 IU/kg in the gluteal muscle of Holstein cows. Although T*max* in the current experiment (1 to 4 h) was comparable to other reports [23,24,38], the C*max* found in our study was remarkably higher than the C*max* reported by Conlon et al. [23]. This may be explained by differences in absorption between different IM injection sites. Indeed, Dubreuil et al. ([24]) reported that a single IM neck injection resulted in a higher C*max* than five consecutive days of IM gluteal injections with the same dosage. Classical pharmacokinetic parameters could not be computed based on the current dataset. It is known that after IM (21,000 IU/kg) administration in cattle, penicillin shows a low volume of distribution and weak protein binding (around 30%). Other pharmacokinetic parameters (C*max*, T*max*, half-life (T_1/2_) and the area under the curve (AUC)) can be found in the scientific literature [34,38] but are beyond the scope of the current article.

One surprising finding of this study was the fact that cows displayed traces or even considerable concentrations of penicillin G in their basal blood samples. Since samples of in-house blank materials were free of penicillin G, inaccuracies in the detection method seem unlikely. Aberrant clearance times after a previous treatment may be an explanation. All cows had calved by CS and at that time were treated by IM injection of procaine penicillin G. However, none of the cows had received any antibiotic treatment for 2 months. Plasma penicillin G concentrations should decline to basal levels within seven T_1/2_, i.e., 70.7 ± 46.2 h [25,26,38]. Medical records of the cows were carefully checked before the trial, but it cannot be excluded that some cows had received a procaine penicillin G treatment beyond our knowledge. Contamination of silages by *Penicillium* mycotoxins [39] is another possible explanation for penicillin traces in untreated animals.

In order to fully understand the efficacy of procaine penicillin G injection, the bacterial population encountered during CS and their susceptibility to penicillin G need to be known in detail [16,17,18]. Only one study has described the bacteria encountered during CS; Mijten et al. [8] isolated *Staphylococci*, *Enterobacteraceae*, *Enterococci*, *Clostridia*, and *Actinomyces* spp. from the peritoneum of 23 cows during CS. Unfortunately, bacteria were not identified up to the species level and their MIC values were not reported. More recently, our research group isolated 32 bacteria strains belonging to 14 bacteria species in 76 peritoneal swabs taken from Belgian blue cows during CS (unpublished results). The predominant germs were *Acinetobacter* spp. (18.7%), *Pseudomonas* spp. (15.6%), *Aerococcus viridans* (12.5%) and *Psychrobacter* spp. (9.4%). Unfortunately, no MIC for penicillin G is available for these bacteria species [40]. Even more complexity is added by the fact that germs identified at the time of CS are not identical to those isolated from infectious complications after surgery [13]. Information on the bacteria encountered during CS, their importance in the pathogenesis of surgical site infections, and their susceptibility to antibiotics should be the focus of further research.

## 5. Conclusions

In conclusion, our results demonstrate that double-muscled cows injected with procaine penicillin G in the neck muscles display a rapid increase in plasma penicillin G concentrations. When injected immediately by the veterinarian after arrival at the farm, plasma penicillin G concentrations 15 min later, at the onset of surgery, will exceed the MIC levels of penicillin G susceptible bacteria throughout surgery. Higher effectiveness of a penicillin injection, i.e., the window of maximal plasmatic concentration coinciding with surgery, can be achieved by injecting 1 to 2 h before the onset of surgery, but this may meet practical difficulties in the field. In any case, a preoperative injection of procaine penicillin G provides higher antibiotic concentrations during surgery than a perioperative or postoperative injection, which is the current practical routine. Whether adapted timing of antibiotic injection also effectively reduces the incidence of infectious complications needs to be confirmed in randomized clinical trials. Additionally, identification of bacteria responsible for infectious complications and their MIC for penicillin G should be the focus of ongoing research.

These data contribute to the evolution towards an evidence-based antibiotic prophylaxis during CS and other bovine surgeries.

## Figures and Tables

**Table 1:** The plasma concentration (ng/mL) of penicillin G at the different sampling times (minute) for each cow enrolled in the trial and the average concentration (Mean ± standard error) reported in our study. Results of a comparable study by Conlon et al. [23] are displayed in the last column.

Sample	Time (minute)	Cow 1 (ng/mL)	Cow 2 (ng/mL)	Cow 3 (ng/mL)	Cow 4 (ng/mL)	Cow 5 (ng/mL)	Cow 6 (ng/mL)	Cow 7 (ng/mL)	Cow 8 (ng/mL)	Cow 9 (ng/mL)	Cow 10 (ng/mL)	Cow 11 (ng/mL)	Cow 12 (ng/mL)	Mean ± SE (ng/mL)	Mean ± SD (ng/mL) [23]
1	0	4.1	62.1	17.7	33.7	3.7	13.3	70.7	39.2	291.9	75.3	22.3	85.3	59.9 ± 22.6 ^a^	ND
2	15	466.1	759.1	644.6	373.9	462.8	501.4	572.8	405.6	1226.9	898.0	846.4	862.4	668.3 ± 73.7 ^b^	820 ± 510
3	30	826.0	1622.0	868.7	668.6	662.7	823.8	633.4	772.1	1790.5	1187.9	1079.7	1115.3	1004.2 ± 108.5 ^c^	880 ± 440
4	45	947.5	1788.7	1132.4	871.8	823.3	1062.2	672.0	716.2	2438.5	1275.5	1478.6	1237.1	1203.6 ± 146.0 ^cd^	800 ± 400
5	60	982.1	2252.5	1181.6	889.3	970.4	1189.4	744.4	931.6	2587.2	1427.9	1453.3	1394.7	1333.7 ± 161.6 ^d^	740 ± 100
6	120	884.5	2882.5	1173.5	840.4	1159.3	1375.3	857.2	1014.3	2241.0	1370.1	1459.5	1262.8	1376.7 ± 175.2 ^d^	770 ± 350
7	240	966.0	1596.4	1035.0	918.9	1213.7	1180.5	1087.9	843.4	1300.3	1660.1	1502.5	1300.7	1217.1 ± 76.7 ^d^	740 ± 420
8	480	769.2	1360.8	667.8	840.2	1061.4	940.4	880.2	705.1	668.7	1442.1	1642.6	1046.4	1002.1 ± 93.2 ^ce^	850 ± 180

a–e values bearing different letters indicate a statistical difference (*p* < 0.05). ND: Not detectable within the limits of the assay. SE: Standard Error, SD: Standard Deviation.

## Data Availability

Data is contained within the article.
